# Untargeted metabolomics of colonic digests reveals kynurenine pathway metabolites, dityrosine and 3-dehydroxycarnitine as red versus white meat discriminating metabolites

**DOI:** 10.1038/srep42514

**Published:** 2017-02-14

**Authors:** Caroline Rombouts, Lieselot Y. Hemeryck, Thomas Van Hecke, Stefaan De Smet, Winnok H. De Vos, Lynn Vanhaecke

**Affiliations:** 1 Ghent University, Faculty of Veterinary Medicine, Department of Veterinary Public Health and Food Safety, Laboratory of Chemical Analysis, Salisburylaan 133, B-9820 Merelbeke, Belgium; 2 Ghent University, Faculty of Bioscience Engineering, Department of Animal Production, Laboratory of Animal Nutrition and Animal Product Quality, Proefhoevestraat 10, B-9090 Melle, Belgium; 3 Ghent University, Faculty of Bioscience Engineering, Department of Molecular Biotechnology, Laboratory of Cell Systems & Imaging, Coupure Links 653, B-9000 Ghent, Belgium; 4 Antwerp University, Faculty of Pharmaceutical, Biomedical and Veterinary Sciences, Department of Veterinary Sciences, Laboratory of Cell Biology and Histology, Groenenborgerlaan 171, B-2020 Antwerp, Belgium

## Abstract

Epidemiological research has demonstrated that the consumption of red meat is an important risk factor for the development of colorectal cancer (CRC), diabetes mellitus and cardiovascular diseases. However, there is no holistic insight in the (by-) products of meat digestion that may contribute to disease development. To address this hiatus, an untargeted mass spectrometry (MS)-based metabolomics approach was used to create red versus white meat associated metabolic fingerprints following *in vitro* colonic digestion using the fecal inocula of ten healthy volunteers. Twenty-two metabolites were unequivocally associated with simulated colonic digestion of red meat. Several of these metabolites could mechanistically be linked to red meat-associated pathways including N’-formylkynurenine, kynurenine and kynurenic acid (all involved in tryptophan metabolism), the oxidative stress marker dityrosine, and 3-dehydroxycarnitine. In conclusion, the used MS-based metabolomics platform proved to be a powerful platform for detection of specific metabolites that improve the understanding of the causal relationship between red meat consumption and associated diseases.

Epidemiological research has consistently demonstrated that a high consumption of red and processed meat is a significant risk factor for the development of several kinds of adenocarcinomas, particulary CRC, cardiovascular diseases and diabetes mellitus^1^,^2^. Several hypotheses have been reviewed to explain the possible underlying working mechanisms, especially in relation to CRC^3^,^4^,^5^. Heterocyclic amines and polycyclic aromatic hydrocarbons are formed during cooking of meat, however chicken also contains high concentrations of HCAs whereas its consumption has not been associated with CRC^3^. A different hypothesis states that the fat content of red meat can contribute to inflammation and intestinal dysbiosis, nevertheless epidemiological research failed to find an association between fat and CRC^4^. Therefore, the main currently accepted hypothesis states that heme iron, present in red meat, can catalyse the formation of N-nitroso compounds (NOCs) and lipid peroxidation products (e.g. malondialdehyde and 4-hydroxy-2-nonenal). NOCs and lipid peroxidation products can induce mutations and specific DNA adducts (e.g. O^6^-carboxymethylguanine), which might eventually lead to the transformation of a normal colon crypt to an abberant crypt focus^3^,^4^,^5^. However, the association between red meat and the development of CRC is highly complex, making it very likely that other mechanisms are involved. More specifically, involvement of the gut microbiome in the development of CRC and other red meat-associated diseases has been proposed^6^. Gut microbiota metabolize food components and produce a plethora of metabolites, some of which may be harmful to the colonic mucosae. For example, nitrogenous substrates may be putrefactively fermented by the gut microbiota and can result in the production of NOCs. This fermentation process is linked to the development and progression of CRC and other bowel diseases^7^. Moreover, diet influences the composition of the gut microbiota. For example, mice on a haem-rich diet had more colonic *Bacteroidetes* and less *Firmicutes*, indicating that a change in the composition of the gut microbiota goes hand in hand with a shift in colonic microbial fermentation products^8^.

Metabolomics comprises the global analysis of all small-molecule metabolites (=endpoints of the gene-protein-metabolite cascade) present within a biological system in a particular physiological state under a given set of environmental conditions^9^,^10^. As a result, metabolomics offers new opportunities to discover how diet influences the metabolic processes of the gut microbiome and elucidate mechanistic links between diet and gut microbiome-associated conditions. To achieve this, nuclear magnetic resonance (NMR) and/or MS may be used in combination with specialized statistical software programs and online databases^11^. With regard to red meat consumption, only urinary metabolomics studies have been published so far, wherein taurine, carnitine, creatinine, acylcarnitines, phophatidylcholine and hexose were identified as red meat intake-associated biomarkers^12^,^13^,^14^.

Up until now, feces or digestive fluids have not been used to investigate the red meat associated metabolome. The main advantage of these biological matrices lies in the presence of colonic and/or microbially formed metabolites allowing a more holistic investigation of the relationship between red meat consumption and risk for disease development, particularly CRC.

In this study, digestive fluids were obtained by simulating the human gastrointestinal digestion of chicken (white meat) versus beef (red meat), whereby the gut microbiota of ten healthy volunteers were used to mimic colonic fermentation. Metabolomic fingerprinting of meat digestion samples was performed by untargeted ultra high performance liquid chromatography coupled to hybrid high-resolution orbitrap mass spectrometry (UHPLC-HR-Q-Orbitrap-MS), an approach that was recently successfully validated for fecal and colonic digestion samples^15^. In addition, chemometric data analysis and further structural elucidation were performed to specifically retain and identify red meat associated colonic metabolites. These metabolites may offer new insights in the mechanisms underlying the causal relationship between the consumption of red meat and associated diseases.

## Results

### Metabolomic fingerprinting of chicken and beef digestion samples

**Figure 1:**
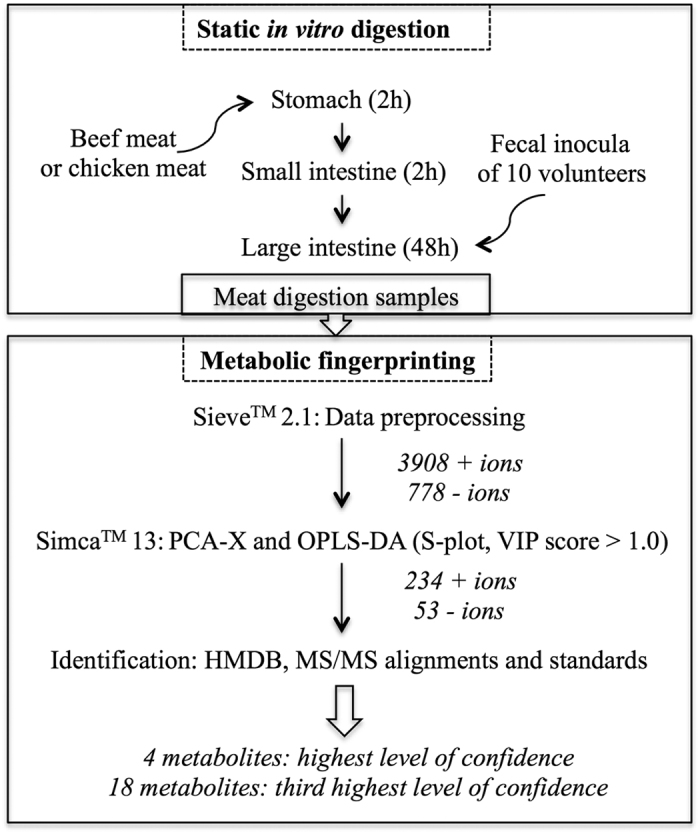
Schematic overview of the study. Static *in vitro* digestions of beef and chicken meat were performed in triplicate. To simulate the colonic fermentation, fecal inocula were obtained from ten healthy volunteers with no history of known gastro-intestinal diseases and/or use of antibiotics at least six months prior to sampling. Sequentially, UHPLC-HR-Q-Orbitrap-MS-based metabolomics was performed on the meat digestion samples, followed by data preprocessing, multivariate statistical analysis and identification of the retained beef-associated metabolites.

**Figure 2:**
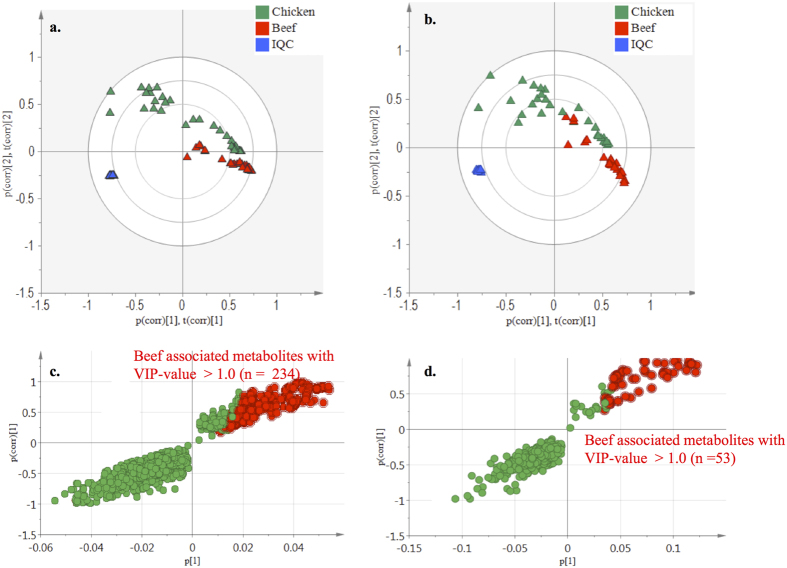
Plots from multivariate statistical analysis of meat digestion samples. (**a**,**b**) Score plots of the PCA-X model for the colonic meat digestion samples after 48 h of incubation in positive (+) and negative (−) ionization mode. The red, green, blue symbols represent the beef, chicken digestion and internal quality control (IQC) samples, respectively. (**c**,**d**) S-plots (OPLS model) for the colonic meat digestion samples after 48 h of incubation in positive (+) and negative (−) ionization mode, wherein each dot represents a metabolite. The metabolites situated in the left lower quadrant are specifically associated with the digestion of chicken and the ones in the right upper quadrant are specifically associated with the digestion of beef (=metabolites of interest). The metabolites marked in red were retained for further analysis.

Untargeted UHPLC-Q-Orbitrap-HRMS metabolomics analysis was performed on *in vitro* colonic (T48) meat digestion samples that were obtained following incubation of red and white meat with the fecal inocula of ten healthy volunteers. In Fig. 1, a schematic overview of the study is presented. Sieve^TM^2.1 data preprocessing resulted in 3908 (+ionization mode) and 778 (−ionization mode) ions. The PCA-X score plots revealed good clustering for the digestion samples according to meat type in both the positive and negative ionization mode (Fig. 2a,b). Datasets were validated by CV-ANOVA (P < 0.01) and the permutation test. The characteristics of the OPLS model were excellent: R^2^X > 0.61, R^2^Y > 0.98 and Q^2^ > 0.97. S-plots were created to retain those metabolites that were specifically associated with the colonic digestion of beef as opposed to chicken meat, whereby the VIP-value was set at >1.0. In total, 234 and 53 red meat associated colonic digestion metabolites were retained in the positive and negative ionization modes, respectively (Fig. 2c,d).

In the positive and negative ionization mode respectively, 75 and 31 associated colonic metabolites were annotated based on accurate mass, using the online available Human Metabolome Database (HMDB)^16^. Next, structural elucidation of these annotated metabolites was performed by means of fragmentation. The online Metfrag tool was used to generate the fragmentation profiles of the possible candidate structures from the HMDB. Twenty-two metabolites were retained (both positive and negative ions) after eliminating those metabolites with no corresponding fragments from the candidate structures. Tables 1 and 2 present statistical significance and identification data of the 22 retained discriminating red meat-associated metabolites, respectively (Tables 1 and 2). Of these metabolites, 3-dehydroxycarnitine and dityrosine were the most important discriminating metabolites between red and white meat, based on VIP-, P-value and area ratios (Table 1).

For 9 of the 22 selected metabolites, commercial standards were obtained. Based on retention time and MS/MS spectra of the corresponding standards, the identity of kynurenic acid, kynurenine, glutarylcarnitine and dityrosine could be confirmed. The remaining 5 metabolites (3-hydroxybutyric acid, (±) furaneol, pantolactone, 4-pyridoxic acid and hexanoylcarnitine) could not be confirmed because of a distinct difference in retention time (>0.20 min) (Table 2). However, the observed divergence in retention time may be due to the fact that the 5 ‘unidentified’ metabolites are in fact isomers of the purchased analytical standards. This is definitely the case for 3-hydroxybutyric acid, because the shift in retention time (<0.50 min) is relatively small, and because of the observed similarities in the obtained MS/MS spectra. Furthermore, in literature, two 3-hydroxybutyric acid isomers are described, namely 2-hydroxybutyric acid and 4-hydroxybutyric acid^17^. For the tentatively identified acylcarnitine (succinylmalonylcarnitine or methylmalonylcarnitine), analytical standards could not be obtained (Table 2), but their identitity was deemed very likely based on the presence of the specific 85.028 Da fragment in its fragmentation profile. In tandem mass spectrometry-based methods for the targeted detection of acylcarnitines, this is the most commonly retrieved fragment^18^. Hexanoylcarnitine could not be confirmed based on the corresponding standard (Table 2), but the specific 85.028 Da fragment was also present here, indicating that this metabolite probably belongs to the acylcarnitine group as well. For 3-dehydroxycarnitine, the MS/MS spectrum complies with the obtained MS/MS data in a study by Tsjujama *et al*.^19^. Moreover, the common 60.081 Da and 87.044 Da fragments matched the Metfrag fragmentation data (Supplementary Fig. 1, Table 2).

**Figure 3:**
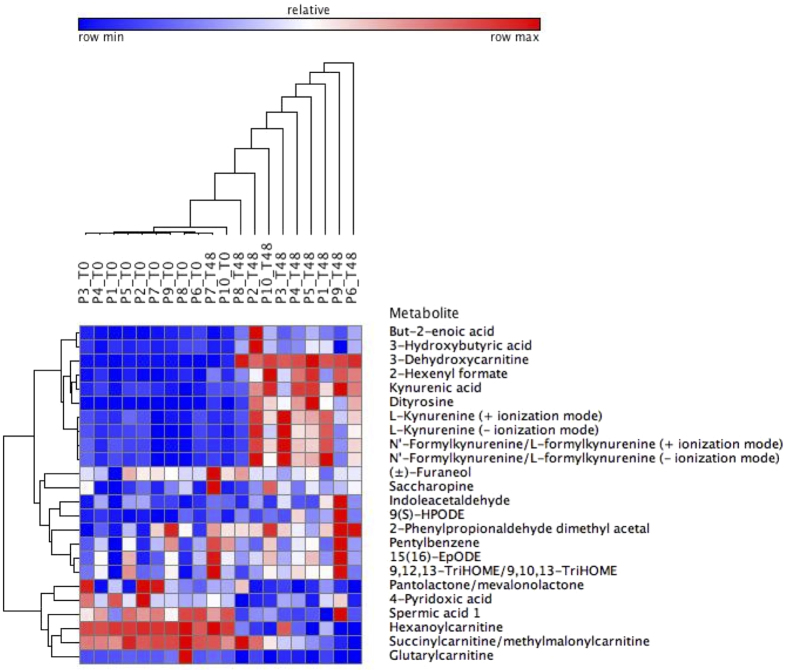
Heat map of red meat-associated metabolites in duodenal (T0) *vs*. colonic (T48) beef digestion samples of 10 participants (P). Hierarchical clustering was performed with Pearson correlation for all participants and annotated metabolites.

The abundance of the retained and (tentatively) identified red meat associated metabolites in the duodenal (T0) *vs*. the colonic digestion samples (T48) are presented in Fig. 3. Clustering (Pearson correlation) of the duodenal samples and colonic digestion samples could be observed (except for P7). On metabolite level, there was clustering between the acylcarnitines, with the exception of 3-dehydroxycarnitine (Fig. 3). The acylcarnitines were more abundant prior to colonic digestion, which indicates degradation in the colon. This was not the case for 3-dehydroxycarnitine, which was more abundant after colonic fermentation (Fig. 3). Additionally, clustering was observed between L-kynurenine, kynurenic acid and N′-formylkynurenine/L-formylkynurenine. Remarkably, dityrosine and 3-dehydroxycarnitine were closely clustered together and were both more abundant in the colon (Fig. 3). 3-Dehydroxycarnitine, dityrosine, kynurenine, L-kynurenic acid and N′-formylkynurenine could be potentially involved in red meat-associated diseases, i.e. CRC^20^,^21^, diabetes mellitus^22^ or cardiovascular diseases^23^ (Table 2). For these 5 metabolites, the MS/MS data are presented in Supplementary Figs 1–5. To further investigate which red meat constituents were involved in the formation of these metabolites, *in vitro* digestions were performed with carnitine and myoglobin (heme). In literature, carnitine has been described as a precursor of 3-dehydroxycarnitine^24^ while kynurenine, kynurenic acid and N-formylkynurenine originate from tryptophan in a chemical reaction that is catalyzed by the heme-containing enzyme indoleamine-2,3-dioxygenase (IDO-1)^25^. Dityrosine is an oxidative stress marker and can therefore result from the Fenton reaction that is catalyzed by heme^3^,^4^,^5^,^26^.

### Targeted metabolomics of carnitine digestion samples

**Figure 4:**
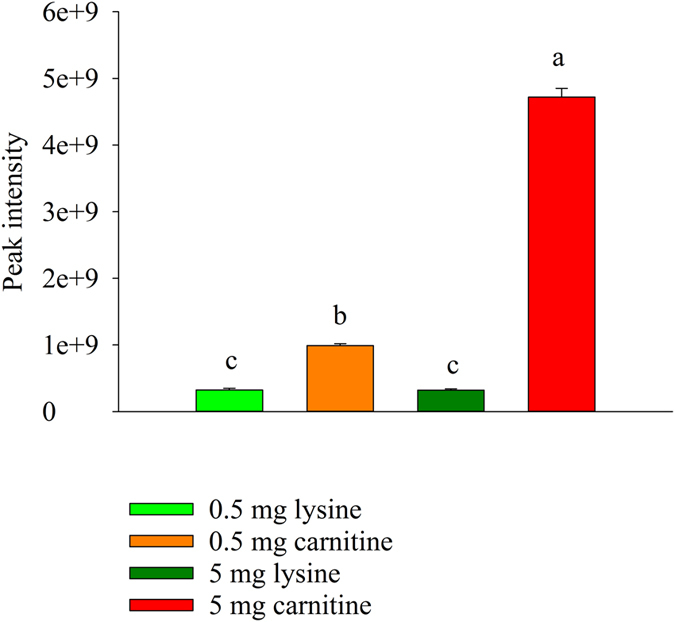
Formation of acylcarnitines in carnitine digestion samples after colonic fermentation. Abundance of 3-dehydroxycarnitine in the carnitine and lysine (0.5 and 5 mg added per flask) digestion samples. Conditions that differ significantly (P < 0.05) are annotated with a different letter and error bars represent the standard deviation (n = 3).

To mechanistically support the formation of the red meat associated acylcarnitines (especially 3-dehydroxycarnitine) during gastro-intestinal digestion, lysine (control) and carnitine, the latter being significantly more abundant in red as opposed to white meat^24^, were subjected to an additional simulated gastro-intestinal digestion. A targeted metabolomics approach measuring 3-dehydroxycarnitine, glutarylcarnitine, the tentatively identified methylmalonylcarnitine or succinylcarnitine and a yet unknown acylcarnitine, was implemented. 3-Dehydroxycarnitine and glutarylcarnitine could be detected in the amino acid digestion samples. 3-Dehydroxycarnitine was significantly (P < 0.0001) more abundant in the carnitine digests in comparison to the lysine digests after colonic fermentation (T48) (Fig. 4). For glutarylcarnitine, no significantly different (P > 0.05) results were obtained.

### Targeted metabolomics of myoglobin digestion samples

**Figure 5:**
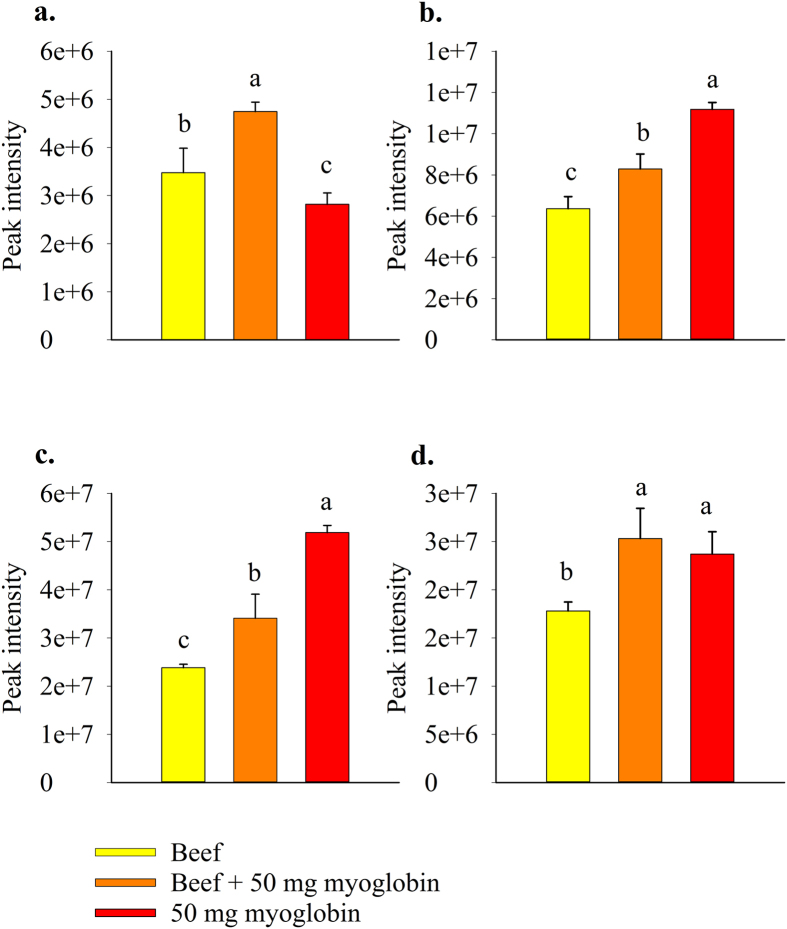
Formation of dityrosine and kynurenine pathway (KP) metabolites in myoglobin digestion samples after colonic fermentation. Abundance of (**a**) dityrosine; (**b**) kynurenic acid; (**c**) N′-formylkynurenine; (**d**) L-kynurenine in beef, beef +50 mg myoglobin and 50 mg myoglobin digestion samples. Conditions that differ significantly (P < 0.05) are annotated with a different letter and error bars represent the standard deviation (n = 3).

To further investigate a possible mechanistic link beween heme iron and kynurenic acid, L-kynurenine, N′-formylkynurenine and dityrosine a gastro-intestinal digestion was set-up in which myoglobin, beef meat or a combination of both were added to the digestion flask. Dityrosine, kynurenic acid, N′-formylkynurenine and L-kynurenine were significantly more abundant in the beef digestion samples combined with myoglobin as opposed to the beef digestion samples without myoglobin (dityrosine: P < 0.0001; kynurenic acid: P = 0.0030; N′-formylkynurenine: P = 0.0005; L-kynurenine: P = 0.0006) (Fig. 5). Dityrosine levels were significantly (P < 0.0001) lower in the myoglobin digestion samples without beef as opposed to the beef containing digestion samples, whereas kynurenic acid (P < 0.0001) and N′-formylkynurenine (P < 0.0001) were significantly more abundant (Fig. 5).

## Discussion

In this study, the fecal inocula of ten healthy volunteers were used to simulate the gastrointestinal digestion of red *vs*. white meat. Untargeted metabolomics by means of UHPLC-Q-Orbitrap technology and multivariate statistical analysis were applied to identify novel leads towards the causal relationship between red meat consumption and associated diseases, particularly CRC.

In total, 22 red meat associated metabolites were (tentatively) identified following the colonic digestion of beef meat, using the HMDB^16^, MS/MS fragmentation profiles and Metfrag. The identities of kynurenic acid, L-kynurenine, glutarylcarnitine and dityrosine were confirmed using a corresponding standard, thus reaching the highest level of confidence for identification according to Sumner *et al*.^27^. For the remaining annotated metabolites, the third highest level of confidence for identification was achieved, because experimental fragmentation profiles and standards were not available for these metabolites^27^. Targeted metabolomics revealed the presence of 3-dehydroxycarnitine in carnitine digestion samples and the presence of dityrosine, kynurenic acid, N′-formylkynurenine and L-kynurenine in myoglobin digestion samples. As such, specific red meat constituents involved in the formation of these metabolites were confirmed.

**Figure 6:**
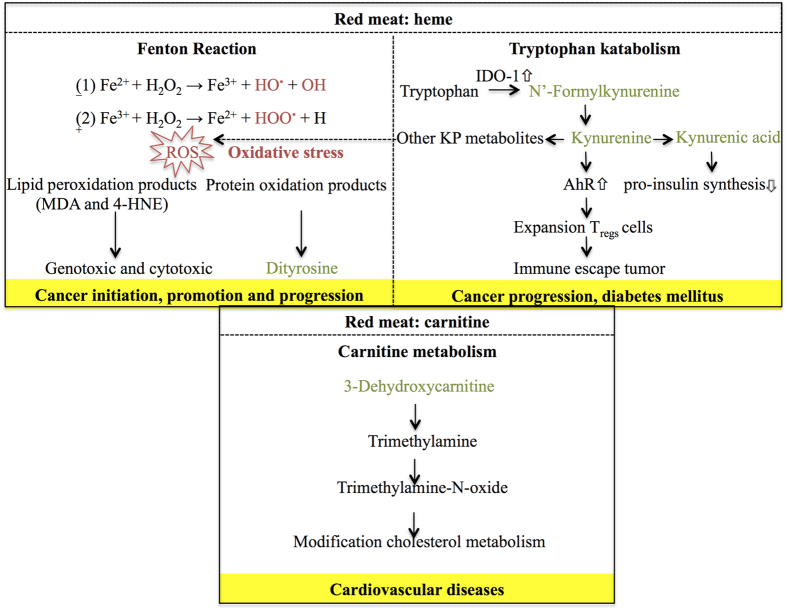
Schematic representation of the involvement of dityrosine, kynurenine pathway (KP) metabolites and 3-dehydroxycarnitine in red meat-associated disease pathways. MDA = malondialdehyde; 4-HNE = 4-hydroxy-2-nonenal; AhR = aryl hydrocarbon receptor.

The results obtained in this study provide relevant new information to elucidate the mechanisms underlying red meat associated diseases, since for several of the retained and annotated red meat associated metabolites (kynurenine, kynurenic acid, N′-formylkynurenine, dityrosine and 3-dehydroxycarnitine) an involvement in the development of CRC, diabetes mellitus and cardiovascular diseases has been conjectured (Fig. 6). Even more, both 3-dehydroxycarnitine and dityrosine were the most discriminating amongst the identified red meat-associated metabolites between the colonic digestion of red as opposed to white meat.

N′-Formylkynurenine, kynurenine and kynurenic acid are intermediates in the catabolism of tryptophan, an essential amino acid that can only be obtained through the diet^28^. Their formation is catalyzed by the heme-containing enzyme indoleamine-2,3-dioxygenase (IDO-1), commonly expressed in all organs^29^. Additionally, active IDO-enzymes have been found in ubiquitous bacteria and fungi such as *Saccharomyces cerevisiae* and *Candida albicans* both prevailing in the human gut^30^,^31^,^32^. IDO-1 activity has been linked to a poor prognosis for different types of cancer, including CRC^33^. Tryptophan is not significantly higher in beef as opposed to chicken meat^34^, implying that other red meat-associated mechanisms must be involved in the formation of the tryptophan catabolites. In this study, the addition of myoglobin to the gastro-intestinal digestive simulations stimulated the formation of the tryptophan catabolites N′-formylkynurenine, kynurenine and kynurenic acid. Therefore, it can be hypothesized that heme catalyzes the formation or increases the activity of IDO-1 by the gut microbiome, since this enzyme is capable of selectively and non-covalently binding to heme^32^. In human tissue, the heme site of IDO-1 is responsible for binding molecular oxygen, which is incorporated in the substrate tryptophan to form N′-formylkynurenine^35^. Nevertheless, literature is lacking about this mechanism in gut microbiota and therefore further research is warranted. Upon addition of myoglobin to beef meat, a decrease in the formation of N′-formylkynurenine and kynurenic acid occurred as opposed to when only myoglobin was added. This implies that other red meat constituents most likely partially inhibit this pathway. Nevertheless, the abundance of these two metabolites was still significantly (P < 0.0001) higher upon addition of myoglobin. Kynurenine is a ligand for the aryl hydrocarbon receptor (AhR) and activation of this receptor leads to expansion of T_regs_ cells, which are responsible for creating an immunosuppressive zone around IDO-1 expressing tumors^25^. Thus, the kynurenine pathway (KP) of tryptophan metabolism is associated with cancer progression, by means of promoting tumor immune escape and growth^20^. In contrast, kynurenic acid can inhibit tumor proliferation, for example, by inhibiting the mitogen activated protein kinase (MAPK) pathway^36^. However, another study demonstrated the association of high levels of kynurenic acid with cognitive impairment, implicating that the abundance of this metabolite determines its biological effect^37^. Additionally, KP metabolites such as 3-hydroxykynurenine and 3-hydroxyanthranilic acid can induce the formation of reactive oxygen species (ROS) under certain micro-environmental conditions, i.e. by reduction of certains metals (e.g. copper and iron) at physiological pH^38^. Given that ROS can damage DNA and induce mutations, this can lead to the transformation of a normal colon crypt to an abberant crypt focus, possibly resulting in the development of CRC later on refs 3, 4, 5. Moreover, KP metabolites have also been linked to diabetes mellitus. More specifically, kynurenic acid was increased in urine of animal models with diabetes type 2 and inhibited pro-insulin synthesis in rats^22^,^39^.

Dityrosine has been proposed as a biomarker of oxidative stress and has been linked to several pathologies. For example, dityrosine concentration levels were significantly higher in low-density lipoproteins isolated from atherosclerotic plaques in comparison to healthy tissue^26^. Also, systemic bacterial infections, inflammatory lung disease, neurogenerative disorders and aging are associated with elevated amounts of dityrosine^40^. The consumption of red meat can lead to oxidative stress through the heme-catalyzed Fenton reaction, resulting in the formation of ROS and lipid peroxidation products^3^,^4^,^5^. These reaction products contribute to the formation of protein oxidation products, whereby tyrosine residues of proteins are oxidized, cross-linked and dityrosine is formed^21^, explaining the increased formation of dityrosine following beef digestion. Additionally, the formation of this metabolite was higher when beef meat was included during the digestion of myoglobin, which demonstrates the importance of other red meat constituents, e.g. proteins. Dityrosine could be of great value as a red meat associated oxidative stress marker, rather than to be causatively linked to the development of CRC. Nevertheless, to the best of our knowledge, this is the first time that dityrosine was proposed as specific for the colonic digestion of red meat as opposed to white meat. Even more, these results are complementary to a study conducted by Rysman *et al*.^41^, wherein the protein oxidation product 4-hydroxyphenylalanine was initially higher in beef as opposed to pork meat and decreased during *in vitro* digestion of meat. The authors suggested that 4-hydroxyphenylalanine was possibly transformed into dityrosine^41^.

Although the red meat digestion resulted in the formation of several acylcarnitines, the digestion of carnitine resulted solely in the formation of 3-dehydroxycarnitine and glutarylcarnitine. However, the peak intensities of this latter metabolite approached the detection limit, therefore giving insignificant results. A logical explanation for the non-production of the other acylcarnitines out of solely carnitine can be found in their presence in the red meat itself. As such, they are initially present in the gastro-intestinal tract and then degraded by colonic bacteria. In general, acylcarnitines are formed during fatty acid metabolism when long-chain acyl groups are transferred to carnitine by coenzyme A in the cytosol. Subsequently, the complexes are transported into the mitochondrial matrix for further oxidation to acetyl-CoA, an important compound in the citric acid cycle^42^. In the utilized *in vitro* digestion model, there is no cellular absorption of carnitine, and therefore no acylcarnitines could be formed and excreted in the colonic fluids. The formation of 3-dehydroxycarnitine was significantly higher in the carnitine as opposed to the lysine digestion samples following colonic fermentation, meaning that this acylcarnitine is microbially formed as opposed to the other acylcarnitines. 3-Dehydroxycarnitine has been identified as an intermediate metabolite in the intestinal bacterial catabolism of L-carnitine, which is more abundant in red meat as compared to white meat, to trimethylamine^24^. The latter can subsequently be converted hepatically into trimethylamine-N-oxide (TMAO), a compound that has been causatively linked to atherosclerosis, through the modification of cholesterol metabolism^23^. It is possible that TMAO is also involved in the onset of CRC, since a recently conducted prospective cohort study revealed that elevated TMAO levels in plasma are associated with higher incidence of CRC in postmenopausal women^43^. Although a direct causal relationship between 3-dehydroxycarnitine, the thereof derived metabolite TMAO, and the development of CRC has not yet been proposed, these metabolites could offer an additional mechanistic explanation for the white meat controversy. In this context, the sole contribution of heme in the red meat-CRC pathway is criticized because the heme content of pork meat is not that different from chicken, but only pork meat is linked to the development of CRC^44^.

A distinction in metabolite profile was observed between the duodenal and colonic digestive fluids, which indicates the active involvement of the colonic microbiota in the formation of the majority of the annotated metabolites and in particular in each of the three hypotheses formulated above. Remarkably, a close clustering was observed between dityrosine and 3-dehydroxycarnitine, with both metabolites being highly abundant in the colon. It has been demonstrated that 3-dehydroxycarnitine is formed by cecal bacteria from the *Bacteroides, Parasutterella* and *Prevotella* genera^45^. Dityrosine, on the other hand results from the enzymatic degradation of oxiditatively modified proteins, suggesting that bacteria with a high proteolytic activity, e.g. *Bacteroides*, could be involved^46^,^47^. The metabolites from the tryptophan pathway are also clustered together and more abundant in the colonic digestion samples. In line with this, the involvement of the gut microbiota in the formation of KP metabolites was illustrated by a study by Clarke *et al*.^48^, during which less kynurenine was produced out of tryptophan in germ free mice as opposed to conventional mice. However, the bacterial mechanisms in tryptophan metabolism remain unclear, but may involve control over degradation of tryptophan^48^.

A clear clustering of meat digestion samples following colonic fermentation was visible and a notable interindividual variation occured among the ten volunteers. The distinction between different meat types most probably relates to their different composition, but might also be influenced by an induced change in microbial composition, i.e. different meats have a different impact on the colonic microbiota and all associated colonic metabolites. In this work, the microbial composition of the fecal inocula of the volunteers was not assessed in order to confirm this hypothesis. However, a study wherein cooked meat samples were incubated with human fecal inocula *in vitro* demonstrated that 48 h of fermentation of chicken meat is associated with a higher number of *Clostridium perfringens*/*histolyticum spp*. and *Bifidobacterium spp*. as compared to 48 h of beef fermentation^49^. This shift is likely to contribute to a different metabolic pattern. The direct impact of diet on the gut microbiome was also shown in a recent study in which an *in vitro* digestion model of the proximal colon (TIM-2 system) was used, demonstrating a shift in the composition of the microbiota and the produced bacterial metabolites following the first 24 h after providing a high carbohydrate diet and a high protein diet^47^. The observed interindividual variation can be explained by genetics and lifestyle factors, i.e. diet, smoking, stress and physical excercise significantly influence the composition of the gut microbiome^50^,^51^,^52^ and metabolome^53^,^54^. Indeed, Weir *et al*.^55^ for example demonstrated that the abundance of certain stool microbiota e.g. *Bacteroides finegoldii*, two *Dialister spp*., and *Prevotella ruminis*, was strongly correlated with increased stool free fatty acids and glycerol, whereas *Ruminococcus spp*. could be associated with an increase in ursodeoxycholic acid^55^.

To conclude, this study provides novel findings concerning the relationship between the consumption of red meat and the development of associated diseases, particularly CRC. We were able to confirm the identity of 3-dehydroxycarnitine, tryptophan catabolites and dityrosine with high confidence and demonstrated the involvement of the red meat constituents carnitine and heme in their microbial formation. Additionally, these metabolites were linked to red meat-associated diseases in literature, which make them promising in further research towards clarifying the responsible red meat-associated mechanisms of disease. The *in vivo* relevance of the newly discovered discriminating red meat-associated metabolites in this study must be further assessed in a large scale follow-up study, during which dietary intake and targeted biomarker analysis of biofluids of healthy controls and patients with CRC or other red meat-associated diseases are performed.

## Materials and Methods

### Meat preparations

Two meat preparations with fresh beef diaphragm (local slaughterhouse) and chicken breast (local butcher) were produced. The meat samples were chopped into cubes of approximately 1–2 cm^3^. Subcutaneous pork fat was added to obtain a total fat content of 20%. The meat preparations were minced using a grinder (omega T-12) equipped with a 10 mm plate, followed by grinding through a 3.5 mm plate. The meat samples were heated in a warm water bath for 30 minutes after the core temperature had reached 90 °C. Finally, the meat preparations were homogenized with a food processor and stored at −20 °C prior to the *in vitro* digestions.

### *In vitro* digestions

#### Collection and preparation of human fecal samples

For the simulation of the colonic digestion, fecal material was obtained from ten volunteers (7 men and 3 women, range 22–75 years). These volunteers had no medical history of known gastro-intestinal diseases and did not use antibiotics for at least six months prior to sampling. Fecal material was processed individually and the fecal inocula were prepared as described previously^44^. All human participants gave verbal informed consent and the experimental protocols were approved by and in accordance with the relevant guidelines and regulations of the government agency for Innovation by Science and Technology (IWT), Belgium that emerged with the Fund for Scientific Research, Belgium in 2016 (FWO-registration number 1S51116N). Submission of an application to the Ethical Committee was not necessary due to the voluntary non-invasive sampling procedure.

#### Simulated gastrointestinal digestion of meat preparations

The *in vitro* simulation of the gastrointestinal digestion consisted of an enzymatic digestion (mouth, stomach and duodenum), followed by colonic fermentation. Preparation of the digestion fluids and their incubations were carried out as described by Van Hecke *et al*.^44^. Digestion fluids were autoclaved before use, with the exception of duodenal juice and pepsine solution (32 mg/100 mL). The latter was sterile filtered through a polyvinylidene fluoride membrane (0.22 μm, 33 mm ∅, Millex, USA). On the day of the *in vitro* digestion, 4.5 g of meat was weighed, after which different digestion fluids were added chronologically and incubated (37 °C). Samples were taken immediately following addition of SHIME (Simulator of the Human Intestinal Micobial Ecosystem) medium and fecal inoculum (duodenal samples, T0) for comparison and after the colonic digestion (T48). Each incubation was performed in triplicate and digestion samples were stored at −80 °C until analysis.

#### Simulated gastrointestinal digestion of carnitine and myoglobin

Stock solutions (5 mg/mL) of carnitine and lysine (control) were prepared in ultrapure water (UP) (Millipore, Brussels, Belgium). Both amino acids were obtained from Sigma-Aldrich (St-Louis, Missouri, USA) and digested *in vitro* by means of the selected fecal inoculum of 1 volunteer. The *in vitro* digestion was performed analogue as for the meat preparations, but instead of 4.5 g meat sample, 1 mL of undiluted stock solution, 1/10^th^ diluted stock solution, or 1 mL of UP water (=control) were added to separate digestions. Additionally, 900 mg of subcutaneous pork fat was included to standardize vet percentage.

Myoglobin, the most important heme-containing protein in mammalian muscle tissue, was obtained from Sigma Aldrich (St-Louis, Missouri, USA). It was added solely or to 4.5 g beef meat at the start of the digestion (50 mg myoglobin per digestion flask). Additionally, digestion flasks with solely 4.5 g beef meat were included as control.

### UHPLC-Q-Orbitrap-HRMS

All digestion samples were centrifuged (21,161 g, 5 min) and the supernatant was filtered through a polyvinylidene fluoride membrane (0.22 μm, 33 mm ∅, Millex, USA). The filtrate was then diluted (1/5) with UP water and transferred to a LC-MS vial. The utilized UHPLC-Orbitrap-MS method was developed, validated and described by Vanden Bussche *et al*.^15^. Injections of an external standard mixture containing *ca*. 300 gastrointestinal metabolites (including amino acids, monocarboxylic acids, phenols, multicarboxylic acids, amines, carbohydrates, polyols, short shain fatty acids, anorganic acids, bile salts and N-compounds) were carried out to assess instrumental stability. Quality control (QC) samples, made from a pool of all individual samples (n = 120), were used for column conditioning (external QC samples) and data normalization (internal QC samples). External QC samples were analyzed in triplicate preceding the batch run and internal QC samples were analyzed in duplicate after each set of 10 samples, which were analyzed in a randomized order. The Q-Orbitrap Exactive^TM^ mass analyzer (Thermo Fisher Scientific, San Jose, USA) was equipped with heated electrospray ionization (HESI II), which was used in polarity switching mode. The instrument was operated in full scan modus with a resolution of 140,000 full width at half maximum (FWHM) at 1 Hz.

### Data analysis

#### Untargeted data analysis

In untargeted metabolomics, different steps are required for data acquisition and analysis, as described by Van Meulebroek *et al*.^56^. The first phase in this general workflow involves data preprocessing with Sieve^TM^ 2.1 (Thermo Fisher Scientific, San Jose, USA). In this study, the data for each ionization mode (+ or −) were processed separately during peak list generation to achieve better model characteristics in Simca^TM^ 13 (see below). First, appropriate parameter settings were applied as follows: beef as the ratio group and reference, frame time width: 0.5 min; *m*/*z*: 53.4–800 dalton, retention time: 0–16 min; *m*/*z* width: 10 ppm; peak intensity threshold: 1,000,000 a.u. for the meat digestion experiment. In the second step, peak alignment, whereby corrections for inherent chromatographic variability were made, was performed after visual evaluation, followed by generating chromatographic peaks (=ions) according to the previous settings. In the final step, a number of discriminative parameters, used to retain only the most relevant ions, were set as follows: ratio (the average ion abundances between samples of different groups): <0.66 or >1.5 and P-value < 0.05. After this, abundances of the residual ions were exported to an excel file. Data normalization was performed by dividing the peak intensity of a particular metabolite in a sample by the mean peak intensity of that metabolite in the following two internal QC samples^15^.

The second phase in the general workflow involves predictive modelling of the retained ions in the statistical program Simca^TM^ 13 (Umetrics, Malmo, Sweden) to select those metabolites that are specific for the digestion of beef as opposed to chicken. For this, the normalized ion abundances were imported in the statistical program. Data were log-transformed to induce normality and scaled by the Pareto method (dividing each variable by the square rooth of the standard deviation), which reduces the relative importance of larger values and partially preserves data structure^57^. An unsupervised principal component analysis (PCA-X) model was created to look for potential outliers. This PCA-X model generates score plots in which data of different biological backgrounds are separated into distinct groups. Samples that cluster together represent a metabolic phenotype^9^. In addition, an orthogonal partial least squares (OPLS) model was created to reveal significant differences between the chicken and beef digestion samples. Herein, the variation is separated into two parts, namely the predictive variation (variation that is common to both X = predictor and Y = outcome) and the orthogonal variation (variation that is not related to Y, i.e. technical and biological factors)^58^. Method-validity was verified with CV-ANOVA (P < 0.01), permutation testing and after inspection of three important model characteristics (R^2^X, R^2^Y and Q^2^Y). CV-ANOVA explains the predictive and orthogonal variation in X, whilst permutation testing explains the total sum of variation in Y. R^2^X and R^2^Y are both goodness-of-fit parameters and Q^2^Y is a goodness-of-prediction parameter. For these three model characteristics, a parameter value >0.5 indicates good model quality^59^. An S-plot was created in the OPLS model to select those metabolites that are specific for the colonic digestion of beef compared to chicken or carnitine compared to lysine. A variable importance in projection (VIP) plot was used to evaluate the importance of a certain metabolite (meat samples: VIP-value >1.0, amino acid samples: VIP-value >0.8).

#### Targeted data analysis

Manual data processing was performed with Xcalibur^TM^ 2.1 (Thermo Fisher scientific, San Jose, USA), during which the *m*/*z* and the retention time of the red meat-associated metabolites were applied in the processing method. Statistical analysis was performed with SAS Enterpruise Guide 7, using two-way ANOVA and Tukey HSD test for post hoc comparisons, whereby a P < 0.05 was considered as statistically significant.

### Metabolite annotation and identification

As a first step in the annotation of the retained red meat associated colonic metabolites, the online Human Metabolome Database (HMDB)^16^ was consulted. To this extent, the accurate masses of the corresponding [M + H]^+^ or [M − H]^−^ were introduced in the database search, and the mass deviation was set at 5 ppm.

As a second step, MS/MS fragmentation profiles were obtained for the metabolites of interest to enable further structure elucidation. To this end, Q-Exactive^TM^ hybrid Quadripole-Orbitrap mass spectrometry (Thermo Fisher scientific, San Jose, USA) was implemented for which the chromatographic separation and ionization settings were the same as described earlier^15^. In addition, parallel reaction monitoring (PRM) with an inclusion list of the metabolites of interest was excecuted, during which all products from a target metabolite are simultaneously monitored under conditions that offer high resolution and mass accuracy^60^. The following MS/MS settings were applied: resolution of 17,500 FWHM, AGC target of 2^e^4, maximum injection time of 40 ms and an isolation window of 2.0 *m*/*z*. Normalized collision energie was set at 20 eV. The online open-source combinatorial fragmenter Metfrag was consulted (http://msbi.ipb-halle.de/MetFrag) to give additional information about possible compound identity. For every possible candidate structure, *in silico* fragmentation was performed using several heuristic rules^57^. Finally, a heat map of the annotated metabolites was constructed with GENE-E software (http://www.broadinstitute.org/cancer/software/GENE-E/index.html).

## Additional Information

**How to cite this article:** Rombouts, C. *et al*. Untargeted metabolomics of colonic digests reveals kynurenine pathway metabolites, dityrosine and 3-dehydroxycarnitine as red versus white meat discriminating metabolites. *Sci. Rep.* **7**, 42514; doi: 10.1038/srep42514 (2017).

**Publisher's note:** Springer Nature remains neutral with regard to jurisdictional claims in published maps and institutional affiliations.

## Supplementary Material

Supplementary Information

## Figures and Tables

**Table 1:** The retained beef associated colonic metabolites after multivariate statistical analysis and identity annotation using the Human Metabolome Database (HMDB).

Metabolites (HMDB)	*m*/*z*	Adduct ion	Δppm	VIP	P-value	Area ratio
But-2-enoic acid	87.04448	H^+^	4.61	1.31	2.03^E-5^	0.49
3-Hydroxybutyric acid	105.05495	H^+^	2.91	1.42	3.32^E-5^	0.45
(±)-Furaneol	129.05450	H^+^	1.08	1.09	3.02^E-5^	0.63
Panto-/mevalonolactone	131.07030	H^+^	0.076	1.20	6.27^E-14^	0.66
2-Hexenyl formate	129.09102	H^+^	0.074	1.52	5.03^E-8^	0.46
3-Dehydroxycarnitine	146.11726	H^+^	2.12	2.37	1.82^E-15^	0.16
Pentylbenzene	149.13216	H^+^	2.28	1.37	2.19^E-9^	0.50
Indoleacetaldehyde	160.07542	H^+^	1.83	1.88	4.29^E-4^	0.17
2-Phenylpropionaldehyde dimethyl acetal	181.12192	H^+^	2.26	1.89	2.55^E-13^	0.38
4-Pyridoxic acid	184.06026	H^+^	1.03	1.49	4.33^E-5^	0.30
Kynurenic acid	190.04955	H^+^	1.80	1.23	8.25^E-6^	0.53
L-Kynurenine	209.09186	H^+^	1.11	1.48	7.99^E-9^	0.46
L-Kynurenine	207.07680	H^−^	3.34	1.62	2.33^E-7^	0.39
Spermic acid 1	218.18567	H^+^	3.01	1.11	9.02^E-6^	0.45
N′-/L-formylkynurenine	237.08652	H^+^	2.05	1.69	4.66^E-8^	0.38
N′-/L-formylkynurenine	235.07198	H^−^	1.82	1.70	2.36^E-7^	0.38
Hexanoylcarnitine	260.18500	H^+^	2.53	1.40	5.55^E-4^	0.015
Succinyl-/methylmalonylcarnitine	262.12766	H^+^	3.35	2.07	4.17^E-9^	0.023
Glutarylcarnitine	276.14352	H^+^	2.42	1.09	6.18^E-5^	0.33
Saccharopine	277.13831	H^+^	4.06	1.41	1.33^E-4^	0.51
15 (16)-epODE	295.22595	H^+^	2.86	1.49	2.66^E-11^	0.48
9 (S)-HPODE	313.23624	H^+^	3.57	1.22	4.89^E-4^	0.52
9,10/12,13-TriHOME	329.23303	H^−^	0.89	1.48	2.05^E-12^	0.50
Dityrosine	359.12442	H^−^	1.16	3.23	8.86^E-8^	0.021

VIP = Variable Importance Plot, P-value = p-value between chicken and beef digestion samples, Area ratio = Average peak intensity of metabolite in chicken digestion samples/average peak intensity of metabolite in beef digestion samples.

**Table 2:** Structural elucidation and identification of the retained beef associated colonic metabolites.

Metabolites (HMDB)	RT		Matching fragments	
	Sample	Standard	Metfrag	Standard
But-2-enoic acid	1.85	NS	1/15	NS
3-Hydroxybutyric acid^a^	1.78	2.21	3/15	8/15
( ± )-Furaneol	2.83	5.71	2/15	ND
Panto-^a^/mevalonolactone	5.02	5.31	1/15	7/15
2-Hexenyl formate	7.28	NS	3/15	NS
3-Dehydroxycarnitine^c^	0.98	NS	3/15	NS
Pentylbenzene	9.81	NS	2/15	NS
Indoleacetaldehyde	4.54	NS	1/15	NS
2-Phenylpropionaldehyde dimethyl acetal	10.42	NS	1/15	NS
4-Pyridoxic acid^a^	6.59	3.06	1/15	ND
Kynurenic acid^b,c^	6.36	6.44	2/15	4/9
L-Kynurenine^b,c^	4.25	4.07	8/15	14/15
L-Kynurenine^b,c^	4.19	4.06	2/15	9/15
Spermic acid 1	0.78	NS	6/15	NS
N′-/L-formylkynurenine^c^	4.76	NS	6/15	NS
N′-/L-formylkynurenine^c^	4.77	NS	6/15	NS
Hexanoylcarnitine^a^	7.87	8.44	1/15	3/15
Succinyl-/methylmalonylcarnitine	1.57	NS	NA	NS
Glutarylcarnitine^b^	2.64	2.69	NA	5/15
Saccharopine	1.18	NS	8/15	NS
15 (16)-epODE	9.89	NS	5/15	NS
9 (S)-HPODE	10.29	NS	6/15	NS
9, 10/12,13-TriHOME	9.89	NS	3/15	NS
Dityrosine^b,c^	2.05	2.18	4/15	3/15

RT = retention time; NS = no standard available; NA = not applicable; ND = not determined.

Standard purchased, but ^a^mismatch in retention time (>0.20 min) or ^b^matching retention time (<0.20 min), ^c^metabolites potentially involved in red meat-associated diseases (e.g. CRC, diabetes mellitus and cardiovascular diseases).
