# Supplementary material for: Untargeted metabolomics of colonic digests reveals kynurenine pathway metabolites, dityrosine and 3-dehydroxycarnitine as red versus white meat discriminating metabolites
Source: Sci Rep. 2017 Feb 14;7:42514. doi: 10.1038/srep42514 (PMC5307356; doi:10.1038/srep42514)

Supporting information

Untargeted metabolomics of colonic digests reveals kynurenine pathway metabolites, dityrosine and 3-dehydroxycarnitine as red versus white meat discriminating metabolites

Caroline Rombouts[1], Lieselot Y. Hemeryck[1], Thomas Van Hecke[2], Stefaan De Smet[2], Winnok H. De Vos[3,4] and Lynn Vanhaecke[1*]

[1] Ghent University, Faculty of Veterinary Medicine, Department of Veterinary Public Health and Food Safety, Laboratory of Chemical Analysis, Salisburylaan 133, B-9820 Merelbeke, Belgium

[2] Ghent University, Faculty of Bioscience Engineering, Department of Animal Production, Laboratory of Animal Nutrition and Animal Product Quality, Proefhoevestraat 10, B-9090 Melle, Belgium

[3] Ghent University, Faculty of Bioscience Engineering, Department of Molecular Biotechnology, Laboratory of Cell Systems & Imaging, Coupure Links 653, B-9000 Ghent, Belgium

[4] Antwerp University, Faculty of Pharmaceutical, Biomedical and Veterinary Sciences, Department of Veterinary Sciences, Laboratory of Cell Biology and Histology, Groenenborgerlaan 171, B-2020 Antwerp, Belgium

***Correspondence**: Prof. Lynn Vanhaecke, Ghent University, Faculty of Veterinary Medicine, Department of Veterinary Public Health and Food Safety, Laboratory of Chemical Analysis, Salisburylaan 133, B-9820 Merelbeke, Belgium

E-mail: Lynn.Vanhaecke@ugent.be

Fax: +32-9-2647492

Figure S1. MS/MS data (+ ionization mode) used for identification of 3-dehydroxycarnitine. MS/MS fragmentation data (PRM) of *m/z* 146.11708.

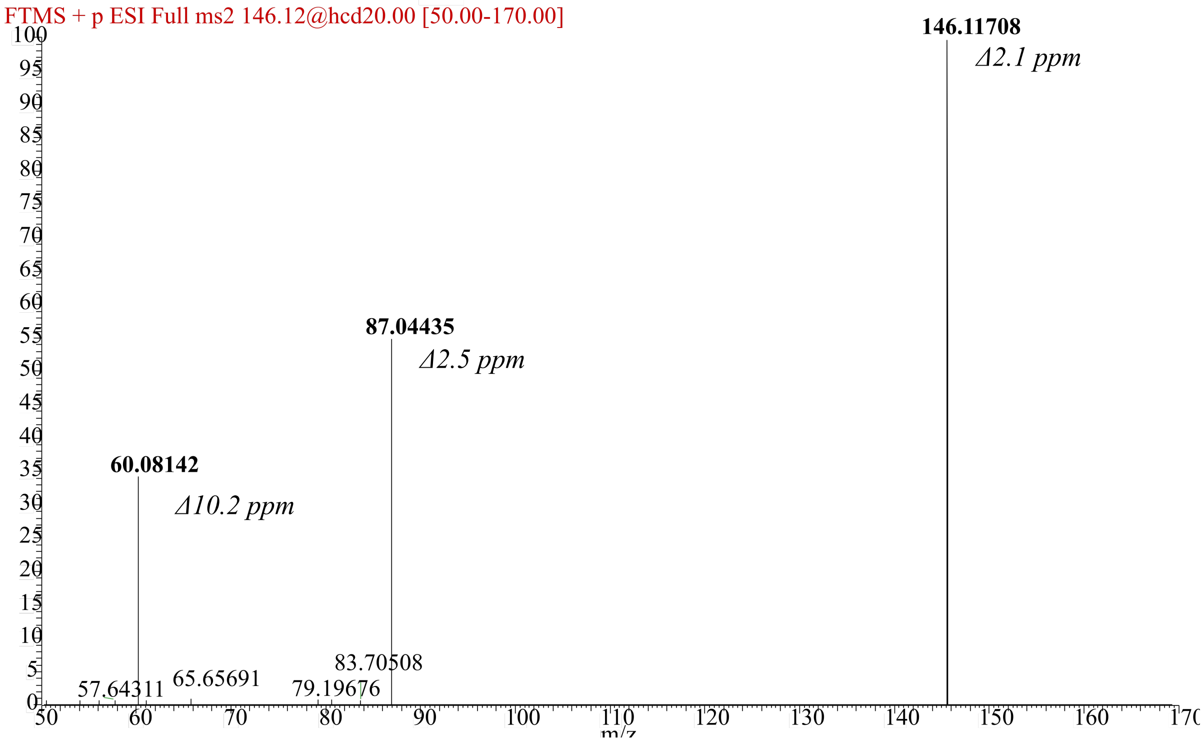

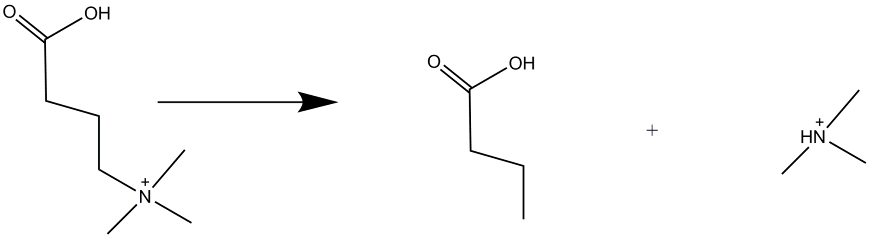

m/z: 146.11756
Chemical Formula: $C_7H_{16}NO_2^+$

m/z: 87.04406
Chemical Formula: $C_4H_7O_2$

m/z: 60.08078
Chemical Formula: $C_3H_{10}N^+$

Figure S2. MS/MS data (+ ionization mode) used for identification of kynurenic acid.

MS/MS fragmentation data (PRM) of *m/z* 190.04919.

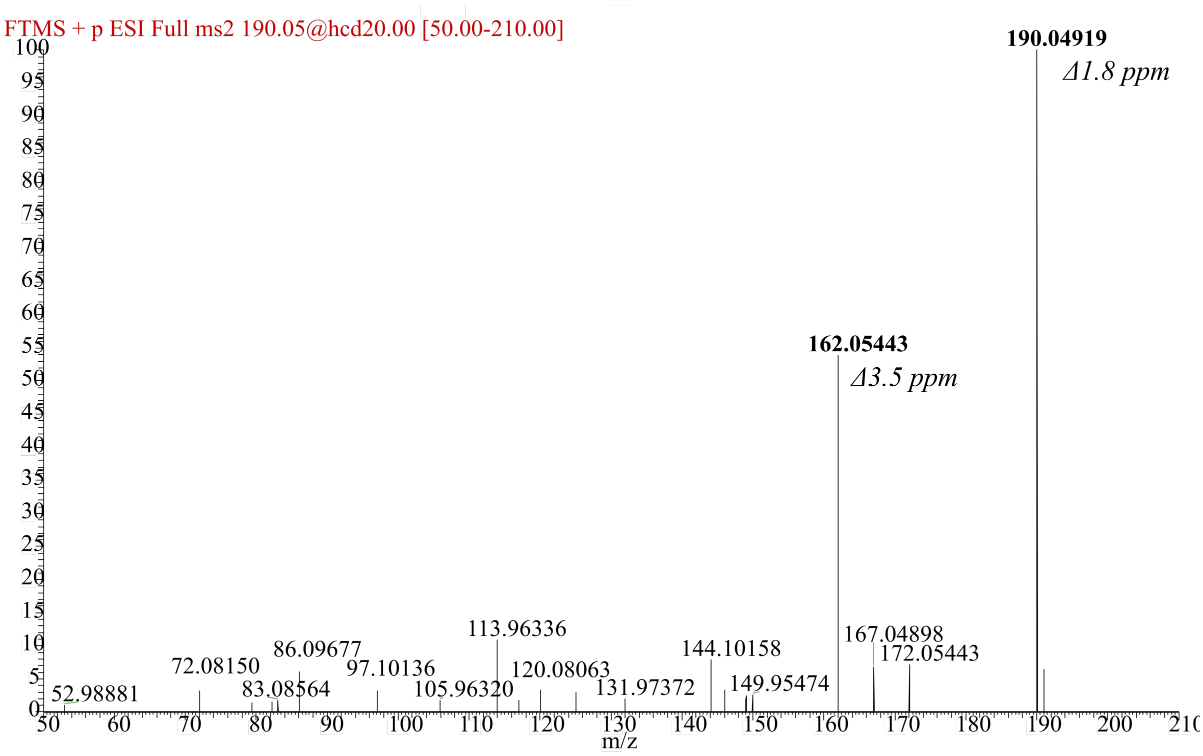

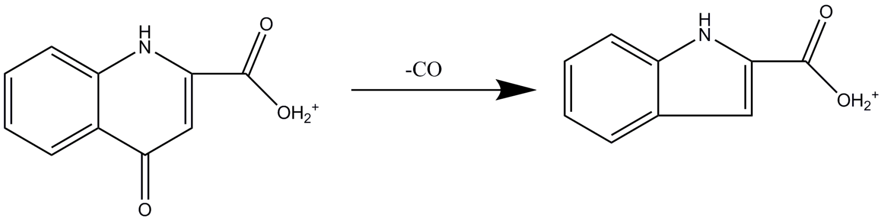

m/z: 190.04987
Chemical Formula: $C_{10}H_8NO_3^+$

m/z: 162.05496
Chemical Formula: $C_9H_8NO_2^+$

Figure S3. MS/MS data (+ ionization mode) used for identification of L-kynurenine.

MS/MS fragmentation data (PRM) of *m/z* 209.09186.

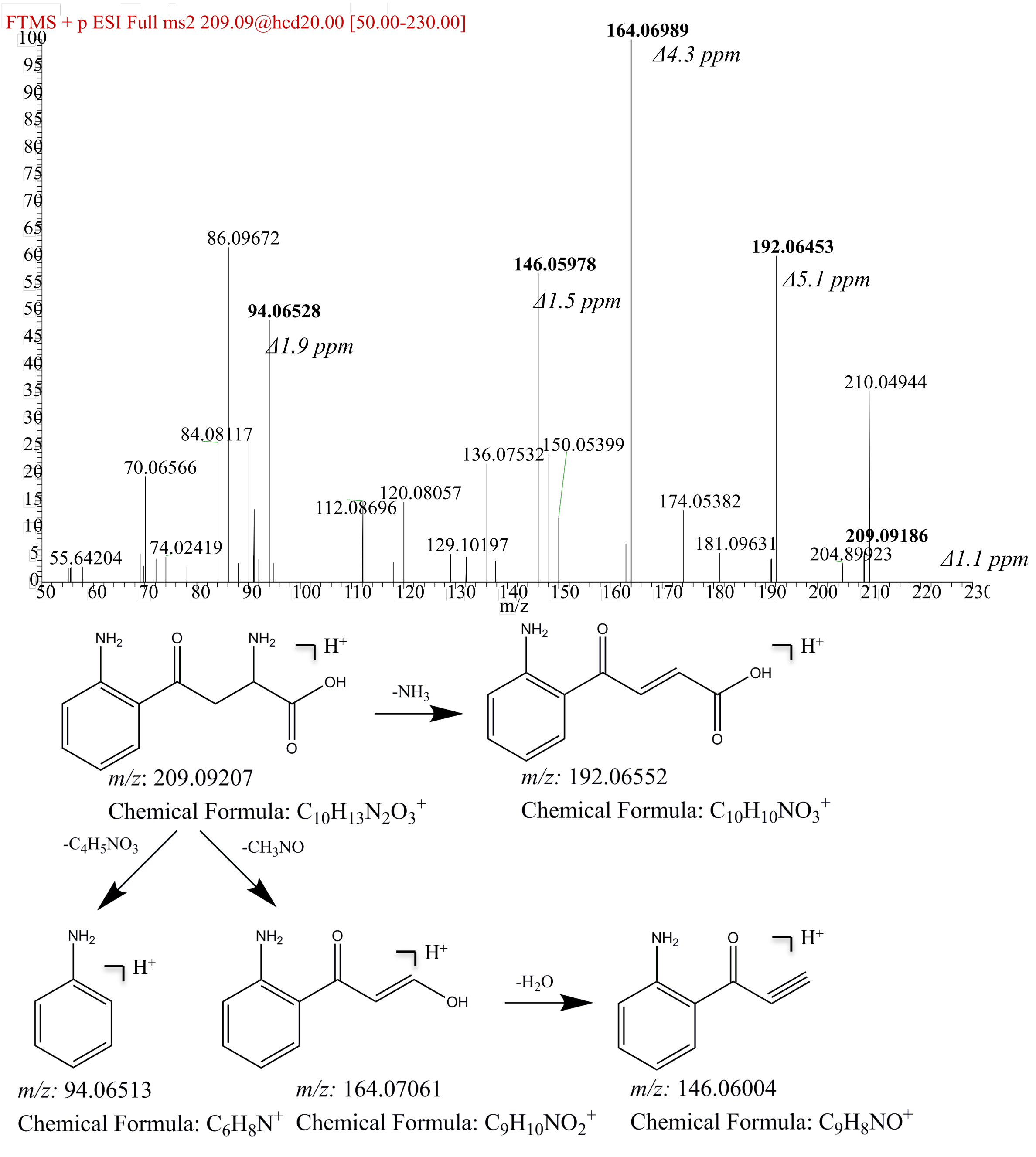

Figure S4. MS/MS data (+ ionization mode) used for identification of N'-formylkynurenine. MS/MS fragmentation data (PRM) of *m/z* 237.08652.

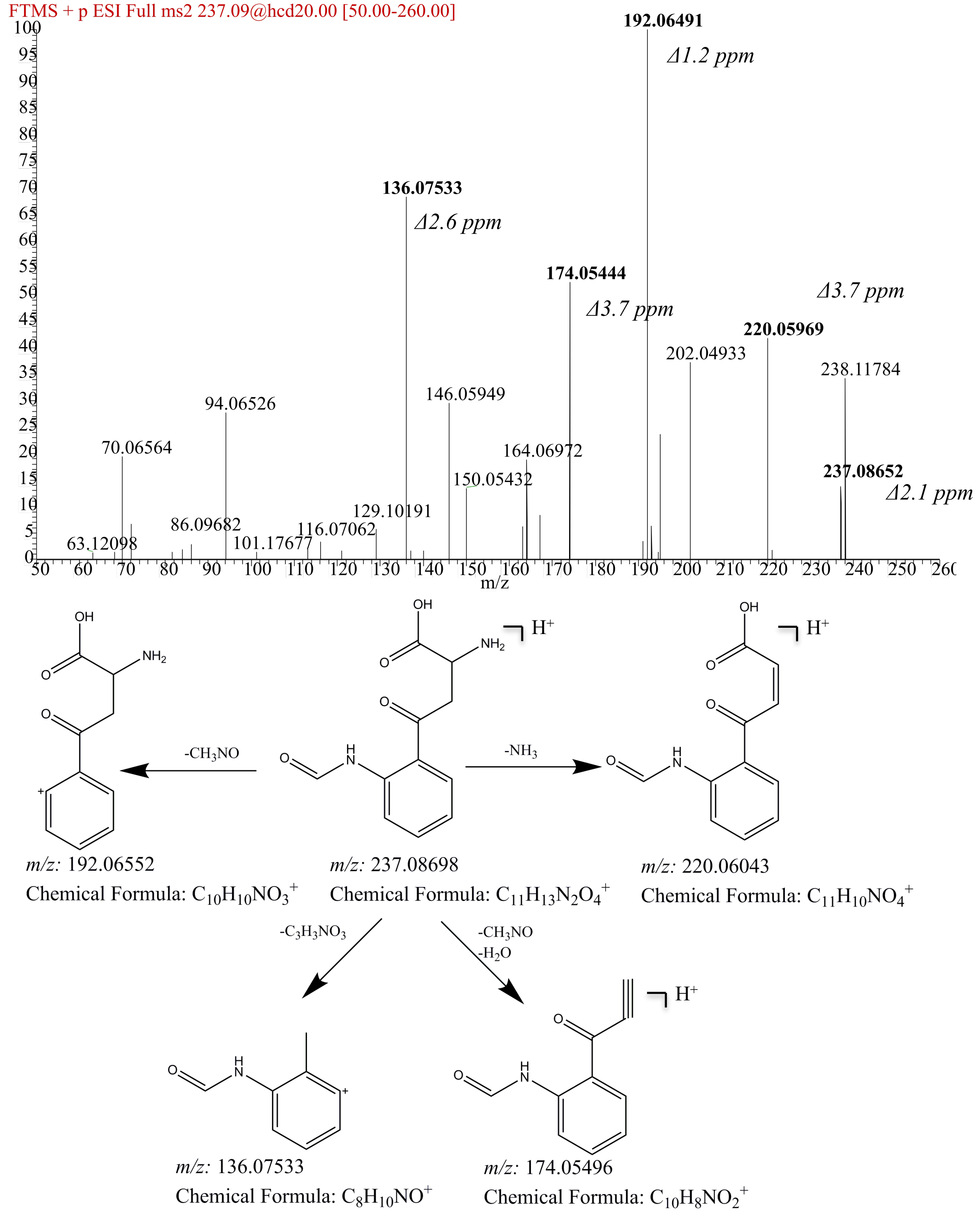

Figure S5. MS/MS data (- ionization mode) used for identification of dityrosine.

MS/MS fragmentation data (PRM) of *m/z* 359.12482.

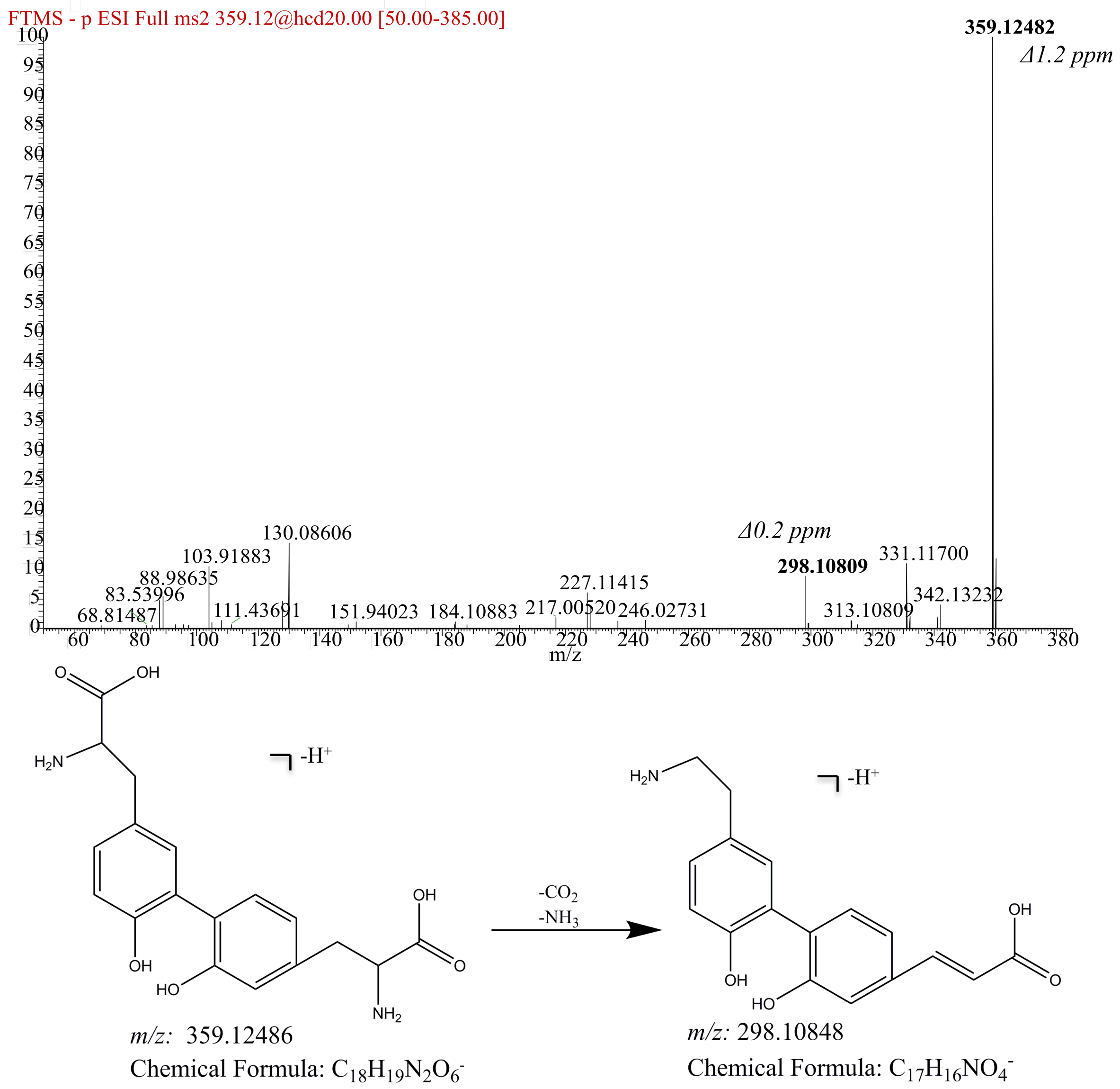

Supplement: Supplementary Information [file srep42514-s1.pdf]
